# Priming Mesenchymal Stem Cells with Lipopolysaccharide Boosts the Immunomodulatory and Regenerative Activity of Secreted Extracellular Vesicles

**DOI:** 10.3390/pharmaceutics16101316

**Published:** 2024-10-10

**Authors:** Aina Areny-Balagueró, Marta Camprubí-Rimblas, Elena Campaña-Duel, Anna Solé-Porta, Adrián Ceccato, Anna Roig, John G. Laffey, Daniel Closa, Antonio Artigas

**Affiliations:** 1 Critical Care Research Center, Parc Taulí Hospital Universitari, Institut d’Investigació i Innovació Parc Taulí (I3PT-CERCA), Universitat Autònoma de Barcelona, 08208 Sabadell, Spain; mcamprubi@tauli.cat (M.C.-R.); ecampanad@tauli.cat (E.C.-D.); aaceccato@tauli.cat (A.C.); aartigas@tauli.cat (A.A.); 2 Centro de Investigaciones Biomédicas en Red de Enfermedades Respiratorias, CIBERES-Instituto de Salud Carlos III, 28029 Madrid, Spain; 3 Institut de Ciència de Materials de Barcelona, ICMAB-CSIC, Campus UAB, 08193 Bellaterra, Spain; asole@icmab.es (A.S.-P.); roig@icmab.es (A.R.); 4 Intensive Care Unit, Hospital Universitari Sagrat Cor, Grupo Quironsalud, 08029 Barcelona, Spain; 5 REMEDI, CÚRAM Centre for Medical Device Research, University of Galway, H91 TK33 Galway, Ireland; john.laffey@universityofgalway.ie; 6 Institut d’Investigacions Biomèdiques de Barcelona, Consejo Superior de Investigaciones Científicas (IIBB-CSIC), 08036 Barcelona, Spain; daniel.closa@iibb.csic.es; 7 Servei de Medicina Intensiva, Corporació Sanitària i Universitària Parc Taulí, 08208 Sabadell, Spain

**Keywords:** acute lung injury, mesenchymal stem cells, extracellular vesicles, LPS priming, immunomodulation, regeneration

## Abstract

**Background:** Mesenchymal stem cells (MSCs)-derived extracellular vesicles (EVs) have been proposed as an alternative to live-cell administration for Acute Respiratory Distress Syndrome (ARDS). MSC-EVs can be chiefly influenced by the environment to which the MSCs are exposed. Here, lipopolysaccharide (LPS) priming of MSCs was used as a strategy to boost the natural therapeutic potential of the EVs in acute lung injury (ALI). **Methods:** The regenerative and immunemodulatory effect of LPS-primed MSC-EVs (LPS-EVs) and non-primed MSC-EVs (C-EVs) were evaluated in vitro on alveolar epithelial cells and macrophage-like THP-1 cells. In vivo, ALI was induced in adult male rats by the intrapulmonary instillation of HCl and LPS. Rats (*n* = 8 to 22/group) were randomized to receive a single bolus (1 × 10^8^ particles) of LPS-EVs, C-EVs, or saline. Lung injury severity was assessed at 72 h in lung tissue and bronchoalveolar lavage. **Results:** In vitro, LPS-EVs improved wound regeneration and attenuated the inflammatory response triggered by the *P. aeruginosa* infection, enhancing the M2 macrophage phenotype. In in vivo studies, LPS-EVs, but not C-EVs, significantly decreased the neutrophilic infiltration and myeloperoxidase (MPO) activity in lung tissue. Alveolar macrophages from LPS-EVs-treated animals exhibited a reduced expression of CXCL-1, a key neutrophil chemoattractant. However, both C-EVs and LPS-EVs reduced alveolar epithelial and endothelial permeability, mitigating lung damage. **Conclusions:** EVs from LPS-primed MSCs resulted in a better resolution of ALI, achieving a greater balance in neutrophil infiltration and activation, while avoiding the complete disruption of the alveolar barrier. This opens new avenues, paving the way for the clinical implementation of cell-based therapies.

## 1. Background

Acute Respiratory Distress Syndrome (ARDS) is a clinical syndrome of diffuse lung inflammation and edema that commonly causes acute respiratory failure [1], representing one of the leading causes of death in intensive care units (ICU) [2]. ARDS can be precipitated by direct or indirect lung damage, which triggers a cascade of inflammatory mediators that can disrupt the alveolar–capillary barrier, resulting in protein-rich pulmonary edema and surfactant inactivation [3]. Importantly, many of these pathways are central to the normal host response to infection or injury, but excessive and diffuse activation can be harmful [1]. ARDS’s biological heterogeneity is a major reason why there is still a lack of a definitive treatment, as no singular underlying pathophysiologic mechanism is present in all patients [4].

Mesenchymal stem cells (MSCs) are low immunogenicity multi-potent cells that can be harvested from different tissues such as bone marrow, umbilical cord blood, adipose tissue or placenta [5]. The therapeutic potential of MSCs-based therapies in the context of acute lung injury (ALI) has been the subject of extensive pre-clinical research revealing its beneficial immunomodulatory, regenerative and antimicrobial effects across a range of animal models and experimental conditions [6]. In this line, we have previously demonstrated that the intratracheal instillation of MSCs reduces pro-inflammatory cytokines, neutrophil infiltration and permeability, and also moderates interstitial edema in an animal model of ALI [7]. Moreover, MSCs safety has been proven in numerous clinical trials [5]. However, their translation from the bench to bedside still has to overcome substantial challenges to be implemented as an ARDS treatment [8].

Extracellular vesicles (EVs) are lipid bilayer particles loaded with several molecules, such as proteins or nucleic acids, that serve as mediators for intercellular communication [9]. In recent years, the EVs secreted by MSCs have become an alternative to live-cell administration, with distinct biological and logistic advantages [10]. The fact that the content of EVs depends on the source cell confers them the ability to mimic the therapeutic effect of MSCs in mitigating inflammatory processes and reducing lung injury [11,12]. There is also evidence suggesting that the composition of EVs in the MSCs secretome is largely influenced by the environments to which the MSCs are exposed [13], and this has become a promising strategy to enhance the therapeutic efficacy of MSCs-EVs [14]. Parameters such as MSCs cellular confluence, cell passage number and oxygen availability, as well as the manipulation of EVs biogenesis biology by priming with cytokines, heparin and the serum content of the medium, can affect both EVs quality and quantity [15,16]. More specifically, several studies have demonstrated that the activation of toll-like receptor 4 (TLR4) of MSCs can change their immunomodulatory function [17,18]. Since the *Escherichia coli* (*E. coli*) lipopolysaccharide (LPS) is one of the best-known ligands of TLR4 and has been demonstrated to activate its intracellular signalling [19], we hypothesized that priming MSCs with LPS would boost the natural therapeutic effect, not only of the MSCs themselves, but also of the EVs they secrete.

Here, we aim to elucidate how LPS priming modifies the therapeutic potential of MSCs-derived EVs in the context of ALI. The present study provides a deeper understanding of the therapeutic effect of MSCs-derived EVs, with a particular focus on their intricate interplay with the immune response and their pivotal role in tissue regeneration.

## 2. Materials and Methods

### 2.1. Isolation, Culture and Preconditioning of MSCs

MSCs were isolated from male Sprague Dawley rats’ femora and tibiae under sterile conditions. The marrow was flushed into a dish containing Dulbecco’s Modified Eagle Medium (DMEM) (Biowest, Nuaillé, France, ref. L0094), supplemented with 10% fetal bovine serum (FBS) (Labclinics, Barcelona, Spain, ref. S181P), 1% penicillin/streptomycin (p/s) (100×, Labclinics, Spain, ref. PS-B), 1% amphotericin B (100×, Labclinics, ref. L0009) and 0.5% L-glutamine (Biowest, France, ref. X0550) and dispersed into a cell suspension. After centrifugation and filtration through 100-μm nylon mesh, the cells were resuspended with DMEM supplemented media and transferred to 75 cm^2^ flasks at a density of 1 × 10^5^ cells/cm^2^. The MSCs were cultured for 72 h at 37 °C and 5% CO_2_, and then non-adherent cells were removed. The MSCs were then washed with phosphate-buffered saline (PBS) 1× and after removal, exosome-depleted FBS (Labclinics, ref. S181M)-supplemented DMEM was added to all flasks with or without *E. coli* 055:B5 LPS (100 ng/mL, Sigma-Aldrich, St. Louis, MO, USA, ref. L2880), to mimic a septic environment, resulting in control MSCs (C-MSCs) and LPS-primed MSCs (LPS-MSCs). The supernatant was collected, and fresh medium was added every 2 days, maintaining the same conditions for 6 days.

### 2.2. Characterization of C-MSCs and LPS-MSCs

Based on the standards set by the International Society for Cellular Therapy [20], the minimum characteristics of MSCs include being plastic adherent in standard culture conditions, expressing stromal surface markers (such as CD44, CD90 and CD105) but lacking hematopoietic cell markers, and having the differentiation potential towards adipogenic, osteogenic and chondrogenic lineages in vitro.

Isolated MSCs proved their ability to adhere to plastic. The immunophenotype of the MSCs was determined by immunofluorescence, evaluating the expression of CD44 (1:10, Abcam, Cambridge, UK, ref. ab24504, rabbit), CD90 (1:1000, Abcam, ref. ab225, mouse) and CD105 (1:100, Abcam, ref. ab156756, mouse). The following secondary antibodies were used to reveal the presence of the primary indicated antibodies: anti-rabbit antibody (1:200, Santa Cruz Biotechnology, Santa Cruz, CA, USA, ref. Sc3917—rTR) and anti-mouse antibody (Santa Cruz Biotechnology, ref. Ics516140—mFITC, 1:100). Cell nuclei were stained with Hoechst (1:10,000, Thermo Fisher Scientific, Waltham, MA, USA, ref. 62249) and the samples were mounted with Fluoromount™ Aqueous Mounting Medium (Sigma-Aldrich, ref. F4680). Positive cells for each marker were counted to assess the percentage of purity using a fluorescence microscope (Eclipse E1000, Nikon, Tokyo, Japan) and ImageJ software (ImageJ 1.40 g; Bethesda, MA, USA).

The MSCs’ capacity to differentiate into osteogenic, chondrogenic and adipogenic lineages was also determined.

Confluent MSCs were cultured at 37 °C and 5% CO_2_ with the respective differentiation media, StemPro™ Osteogenesis, Chondrogenesis and Adipogenesis Differentiation Kit (Thermo Fisher Scientific, ref. A1007201, A1007101, A1007001). The media was changed every 48 h. After 7 days, cells were incubated in Oil Red O solution to stain the intracellular lipid-rich vacuoles and then the cells were stained with Mayer’s hematoxylin and rinsed with current water. After 14 days, the chondrocytes were incubated in 1% Alcian Blue solution and then removed with HCl. After 21 days, the osteocytes were stained with 2% Alizarin Red S solution and washed with distilled water. Undifferentiated MSCs were stained using the same protocols described below. The samples were mounted with DPX Mounting Media (Sigma-Aldrich, ref. 06522) and imaged using a Nikon Eclipse Ti microscope.

### 2.3. C-EVs and LPS-EVs Isolation and Characterization

EVs were isolated as described by Thery, C et al. [21], using differential centrifugations. Previously collected MSC culture media from C-MSCs and LPS-MSCs were centrifuged at 2000× *g* and 10,000× *g* for 10 and 30 min, respectively, at 4 °C. The 10,000× *g* supernatant was filtered through a 0.22 µm filter and ultracentrifuged at 110,000× *g* for 2 h and 20 min. The pellets were resuspended in sterile PBS 1× to obtain two different pools of EVs: C-EVs and LPS-EVs.

The concentration and the mean size of the isolated EVs were analyzed by nanoparticle tracking analysis (NTA) in a NanoSight LM10 machine (NanoSight, Amesbury, UK) by the ICTS “NANBIOSIS” at the ICMAB-CSIC. All the parameters of the analysis were set at the same values for all samples and three 1-minute-long videos were recorded in all cases. The background was measured by testing filtered PBS 1× (Sigma-Aldrich, ref. P4707), which revealed no signal. The protein concentration of each pool of EVs was quantified by Bradford Protein Assay (Thermo Fisher Scientific, ref. 23200).

The quality of the EVs samples was also verified by cryo-transmission electron microscopy (cryo-TEM-2011 operating at 200 kV, UAB service) [22]. The presence of specific EV membrane proteins such as, TSG101 (1:500, ref. 14497-1-AP) and ALIX (1:500, ref. 12422-1-AP), and a multi-pass transmembrane protein, CD81 (1:500, ref. 10630D) [23], was detected by Western Blot analysis. The absence of Calnexin (1:1000, ref. 0427-2-AP) (ProteinTech, Rosemont, IL, USA) was used as a negative control for other cellular compartment vesicles.

### 2.4. Assessment of MSCs-Derived EVs’ Therapeutic Effect In Vitro

Scratch wound assay: Human Pulmonary Alveolar Epithelial cells (HPAEpiC) (Innoprot, Derio, Spain, ref. P10556) were grown until confluence in a 12-well plate with alveolar epithelial cell medium (Innoprot, Derio, Spain, ref. P60102), supplemented with 1% p/s, 1% EpiCGS and 2% FBS, and a single scratch wound was made in each well with a 1000 µL pipette tip. The wells were aspirated, rinsed with PBS 1× and re-fed with fresh media, followed by 1 × 10^4^ of C-EVs or LPS-EVs/cell. Scratch wounds were imaged at 0 h and 24 h after the treatment by a phase contrast microscope (Leica Microsystems, Wetzlar, Germany), and wound width was assessed by measuring pixel distance across the wound using ImageJ software (ImageJ 1.40 g). The basal wound closure (control group) was considered the 100%.

Cell proliferation assay: The CellTiter 96^®^ AQueous One Solution Cell Proliferation Assay (Promega, Madison, WI, USA, ref. G3582) was used. HPAEpiC were seeded at a concentration of 1 × 10^4^ cells/well in a 96-well plate and they received 1 × 10^4^ of C-EVs or LPS-EVs/cell. After 24 h, the cells were washed with PBS 1× and incubated with the CellTiter 96^®^ AQueous One Solution Reagent containing a tetrazolium compound [3-(4,5-dimethylthiazol-2-yl)-5-(3-carboxymethoxyphenyl)-2-(4-sulfophenyl)-2H-tetrazolium, inner salt; MTS] for 1 h at 37 °C in a humidified cell culture incubator. The amount of formazan generated was quantified by measuring the absorbance at 490 nm on a microplate reader. The basal cell viability (control group) was set as the 100%.

Immunomodulatory assay: The human monocytic cell line, THP-1 (ATCC, Manassas, VA, USA, ref. TIB-202), was seeded at 1.5 × 10^5^/well in a 24-well plate and differentiated to macrophages by exposing them to phorbol 12-myristate 13-acetate (PMA) (100 ng/mL, Sigma-Aldrich, ref. P8139) diluted in Roswell Park Memorial Institute (RPMI) 1640 medium (Biowest, ref. L0501) supplemented with 10% inactivated FBS, 1% p/s, 1% amphotericin B and 0.5% L-glutamine for 48 h at 37 °C. Five hours before the infection, differentiated cells were primed with fresh RPMI 1640 media containing LPS (2 μg/mL) to polarize them into a pro-inflammatory M1 phenotype. Negative controls received the same media without LPS.

*Pseudomonas aeruginosa* (*P. aeruginosa*, PA) (strain PAO1) (IDIBAPS, Barcelona, Spain) was seeded on blood agar plates 3 days before the infection of the cells. After 24 h, seeding through the streak plate method was performed. Pre-inoculum was prepared by picking 6 colony-forming units (CFU) of *P. aeruginosa* and inoculating them in 10 mL Luria Broth (LB) medium (Thermo Fisher Scientific, ref. 10855001) in a 50 mL falcon for 16 h at 37 °C and 200 rpm. After this time, 2 mL of pre-inoculum was added to 100 mL of LB medium in a 250 mL bottle. The inoculum was incubated at 37 °C and 200 rpm until an absorbance of >0.4 was achieved (measured with an OD of 600 nm), which corresponds to the log phase (≈10^9^ CFU *P. aeruginosa*/mL). The inoculum was then centrifuged for 15 min at 1500× *g* at RT.

Five hours after the LPS priming, the THP-1 cells were washed twice with PBS 1×. *P. aeruginosa* was added with fresh RPMI 1640 media with a multiplicity of infection (MOI) of 1:50. The cells were incubated with the bacteria for 1 h at 37 °C. Then, the cultures were washed 3 times with PBS 1× containing p/s 5× and were re-fed with fresh media followed by 1 × 10^4^ of C-EVs or LPS-EVs/cell. After 24 h, the cells were recollected with TRizol^®^ reagent (Thermo Fisher Scientific, ref. 15596026) and RNA was extracted by chloroform-isopropanol isolation. The expressions of the housekeeping RPL37a and interleukin (IL)-1β, IL-6, IL-8, CD86 and CD206 mRNA were evaluated by real-time quantitative reverse transcription polymerase chain reaction (RT-qPCR) using KAPA SYBR^®^ FAST One-Step Kit (Sigma-Aldrich, ref. KK4652) and the corresponding primers (Supplementary Table S1). The relative expression of target genes was normalized to RPL37a expression by the ΔC(t) formula.

### 2.5. In Vivo MSCs-Derived EVs’ Therapeutic Evaluation

Animals: Male Sprague Dawley rats (Charles River, France) weighing 200–225 g at the beginning of the experiment were used. They were kept under controlled environmental conditions (temperature and relative humidity), a 12:12 h light–dark cycle, with enrichment material placed inside the cages and free access to food and water. This study was performed in accordance with the European Community Directive 86/609/EEC and Spanish guidelines for experimental animals, approved by the Animal Research Ethics Committee of the Autonomous University of Barcelona and the Animal Experimentation Committee of the Generalitat de Catalunya (CEEAH 6340).

Development of ALI pre-clinical model and EVs administration: ALI was induced as in our previous study [24]. The animals received an intratracheal instillation of 300 µL of HCl (0.1 M at pH = 1.4) and, 2 h later, an intratracheal instillation of the endotoxin LPS (30 µg/g of body weight) dissolved in 500 µL of saline (0.9% NaCl) under sevoflurane anesthesia (Piramal, Voorschoten, The Netherlands). Nine hours after the endotracheal LPS administration (or saline, in the case of control animals), recipient animals were administered with C-EVs or LPS-EVs intratracheally by the trans-oral route under sevoflurane anaesthesia. Each animal received a single bolus of 1 × 10^8^ particles (determined by the NTA) suspended in 300 μL of sterile saline. The control groups received the same volume of saline. During the experiment, the animals were continuously supervised and body weights were recorded every 24 h. Animals were anesthetized intraperitoneally with ketamine (90 mg/kg) and xylazine (10 mg/kg), and were exsanguinated from the abdominal aorta at 72 h after the induction of ALI. The lungs were removed and weighed. Bronchoalveolar lavage (BAL) was either performed in unilobular and multilobular lungs, while histology was conducted in the unilobular lung and the multilobular lung was frozen for lung tissue analysis. The exact number of animals used for each analysis is indicated in the figure legends.

Bronchoalveolar lavage technique and analysis: The BAL was performed by washing 5 times the unilobular lung with 5 mL of saline.

Differential and total cell count analysis: After treating the total BAL cells with ammonium chloride potassium (ACK) (Gibco, Waltham, MA, USA, ref. 11509876), they were Fc blocked with CD16/CD32 antibody (1:50, BioLegend, San Diego, CA, USA, ref. 101302) and then stained with the antibody mix (Supplementary Table S1) at 4 °C in the dark for 30 min. The cell pool was analyzed by a FACSCANTO II cytometer with 3 lasers (IDIBAPS platform service) for measurement and classification of different cell leuckocyte subsets. CountBright™ Absolute Counting Beads (Thermo Fisher Scientific, ref. C36950) were added to each sample for absolute cell count. Data were analyzed by using FlowJo (version 10.8.1). Cell subset populations were gated as followed after selecting singlets (Supplementary Table S2, Supplementary Figure S1): total myeloid cells, CD45+ and CD11b+; classical monocytes, CD45+, CD11b+ and His48+; and neutrophils, CD45+, CD11b+ and His48++ with high granularity (side scatter (SSC-A)).

The differential cell count in BAL was also evaluated in cytospin preparations stained with Diff-Quick Kit (Panreac Quimica SAU, ref. 254101, 253999, 253998), according to manufacturer’s protocol. Monocytes/macrophages, neutrophils and lymphocytes were counted considering their cellular and nuclear morphology. Monocytes: bi-lobed nuclei, which became more rounded following differentiation; neutrophils: segmented, multi-lobed nuclei, containing between two and five lobes; lymphocytes: rounded nuclei with less cytoplasm volume [25]

Total protein quantification and alveolar macrophages obtaining: The BAL was centrifuged for 5 min at 500× *g* and the supernatant of the BAL was collected for total protein measurement using the bicinchoninic acid (BCA) protein assay (Pierce™ BCA Protein Assay Kit, ThermoFisher Scientific), according to the manufacturer’s protocol. The pellet was resuspended in RPMI 1640 medium supplemented with 10% FBS, 5% L-glutamine, 1% p/s and 1% amphotericin B, and was incubated at 37 °C for 1–2 h. Adhered cells corresponded to alveolar macrophages (AM), which were rinsed with PBS 1× twice and their purity was confirmed by microscopy. AM were collected for RNA extraction.

Histological studies: The unilobular lungs were embedded in paraffin and 4 μm-thick histological sections were obtained. They were stained with hematoxylin-eosin (H&E) and evaluated under bright field microscopy (Nikon Eclipse Ti microscope). The images were evaluated using the ImageJ software (ImageJ 1.40 g; W. Rasband, NIH). Whole lung sections were analyzed at 2.5× magnification to assess the overall presence and extent of damage. The lung injury score (LIS) was quantified blindly by three investigators using Supplementary Table S3 [26]. The LIS was obtained by the sum of each of the five independent variables (hemorrhage, peribronchial infiltration, interstitial edema, pneumocyte hyperplasia and intra-alveolar infiltration) at 10× and was normalized to the number of fields evaluated. The resulting injury score was a value between 0 and 10 (both inclusive) [27].

Inflammation in lung homogenates and AM: Total RNA from lung tissue and BAL macrophages were extracted using TRizol^®^ reagent (Thermo Fisher Scientific, ref. 15596026) and chloroform-isopropanol isolation. The mRNA of pro-inflammatory cytokines (IL-1β and IL-6), chemoattractant mediators (CCL2 and CXCL-1) and M2 phenotype markers (Arginase-1 (Arg-1) and Mannose receptor (MR)) were quantified by RT-qPCR and the corresponding primers (Supplementary Table S1). The relative expression of target genes was normalized to the housekeeping GAPDH expression by the ΔC(t) formula.

Measurement of myeloperoxidase activity in lung tissue: Myeloperoxidase (MPO) was measured with 3,3′,5,5′-Tetramethylbenzidine (TMB) as a substrate. Tissue samples were homogenized with hexadecyltrimethylammonium bromide and monopotassium phosphate (KH_2_PO_4_). Homogenates were disrupted for 120 s using a 3D Minilys tissue homogenizer (Bertin Technologies, Montigny le Bretonneux, France) and submitted to three cycles of snap freezing in dry ice and thawing before a final 60 s sonication. The samples were incubated at 60 °C for 2 h to eliminate the activity of unspecific peroxidases and then spun down at 15,000× *g* for 15 min. The supernatants were collected for MPO quantification. To cease the reaction, a solution containing KH_2_PO_4_ and hydrogen peroxide (H_2_O_2_) was added. The enzymatic activity was assessed spectrophotometrically at 620 nm. The standard curve for calculating the MPO concentration in each sample was prepared with horseradish peroxidase (HRP). Data were presented as milliunits of MPO/total grams of tissue protein (mU/g) (quantified by BCA Protein Assay).

### 2.6. Statistics for In Vitro and In Vivo Studies

All the data were analyzed using GraphPad Prism 9 software and expressed as the mean ± standard error of the mean (SEM). A one-way ANOVA with Tukey’s multiple-comparison test was applied to compare more than two groups and a two-way ANOVA followed by Dunnett’s multiple comparison test was used to analyze data with more than one variable. Data showing high dispersion were log2-transformed to ensure the assumptions of the linear model. All statistical tests were two-sided and a *p*-value (*p*) ≤ 0.05 was considered statistically significant. The number of the samples (*n*) analyzed in each parameter are specified in the figure captions.

## 3. Results

### 3.1. Characterization of Isolated MSCs

**Figure 1:**
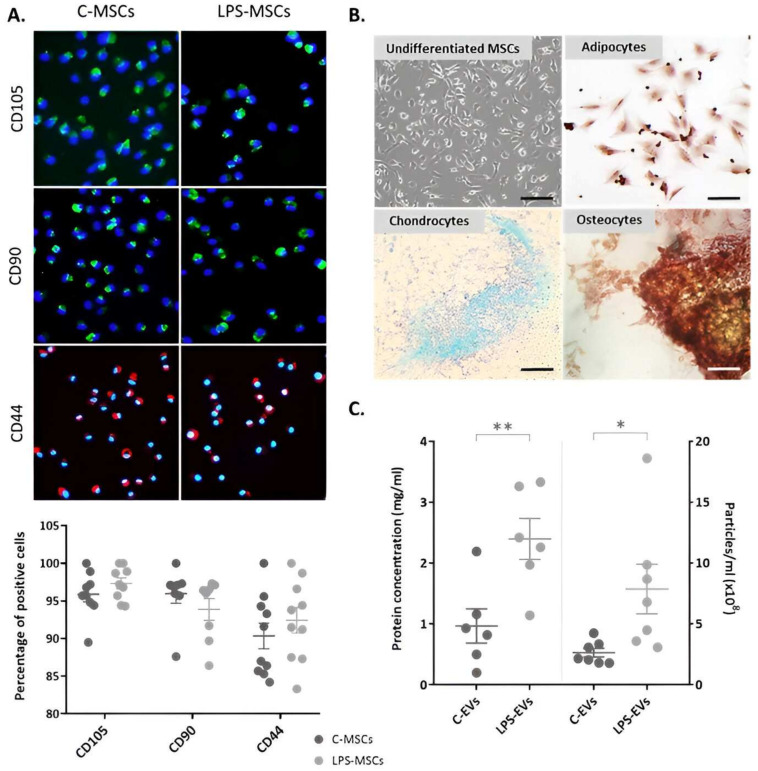
Characterization of C-MSCs and LPS-primed MSCs isolated from rat bone marrow. (**A**) Immunophenotype of C-MSCs and LPS-MSCs, staining CD105 (FITC, green), CD90 (FITC, green) and CD44 (Texas Red, red) markers. Quantification of the percentage of positive cells for each marker (*n* = 7–11). The nuclei of the cells were stained with Hoechst (UV light, blue), 20× magnification. Scale bar: 100 µm. (**B**) Representative images of undifferentiated MSCs and MSCs differentiation towards adipogenic, osteogenic and chondrogenic lineages in vitro. Magnification: 10×. Scale bar: 500 µm. (**C**) Quantification of the EVs-like particles (**right**) (*n* = 5) and protein (**left**) (*n* = 7) concentration in C-MSCs and LPS-MSCs media. Data are presented as mean ± SEM (*n* = 3); * *p* < 0.05; ** *p* < 0.01. Abbreviations: extracellular vesicles, EVs.

C-MSCs and LPS-MSCs were positive for specific markers of mesenchymal cell lineages such as CD105, CD90 and CD44, showing a purity of 93.1 ± 3% and 94.2 ± 2%, respectively. The treatment with LPS did not affect the expression of any of these markers (Figure 1A). They presented plastic adherence, exhibited a spindle-shaped morphology and were capable of differentiating into adipocytes, chondrocytes and osteocytes (Figure 1B).

Regarding the secretory function of the MSCs, LPS-MSCs presented a significantly higher number of EVs-like particles (*p* = 0.026) and protein concentration (*p* = 0.008) in their culture media, demonstrating an enhanced paracrine activity compared to C-MSCs (Figure 1C).

### 3.2. Characterization of MSCs-Derived EVs

**Figure 2:**
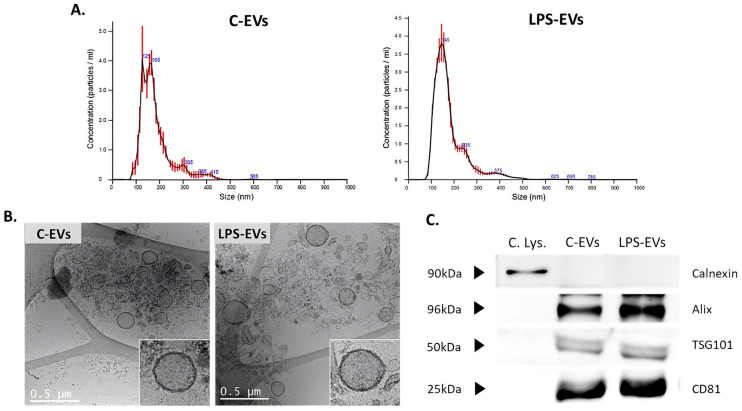
Characterization of C- and LPS-MSCs-derived EVs. (**A**) Representative particle size distributions of C-EVs and LPS-EVs’ pools by Nanosight analysis. (**B**) Representative cryo-TEM images of C-EVs and LPS-EVs, 12kX magnification. Scale bar: 0.5 µm. (**C**) Detection of Alix, TSG101 and CD81 surface markers in C-EVs and LPS-EVs by Western Blot analysis. Detection of Calnexin only in cell lysate samples as negative control. Abbreviations: cell lysate, C. Lys.; extracellular vesicles, EVs.

No significant differences in the size, morphology or expression profiles of specific EVs’ markers between EVs secreted by C-MSCs and LPS-MSCs were found. Size distribution evaluated by NTA indicated that the average size of C-EVs was 170 nm (average of mode size = 138 nm) and 181 nm (average of mode size = 146 nm) for LPS-EVs (Figure 2A), compatible with small EVs [23]. This was supported by cryo-TEM images, which also showed that the morphology and the size of the components that were isolated were consistent with the characteristics of small EVs (Figure 2B). Finally, Western Blot analysis confirmed the presence of EVs-specific surface markers such as Alix, TSG101 and CD83 in both pools, sustaining that our samples contained mainly EVs. In addition, the absence of calnexin, an endoplasmic reticulum marker, confirmed that the potential contamination of the EVs populations with non-vesicular co-isolated components was minimal (Figure 2C).

### 3.3. LPS-EVs Accelerate Wound Healing and Cell Proliferation versus C-EVs In VItro

**Figure 3:**
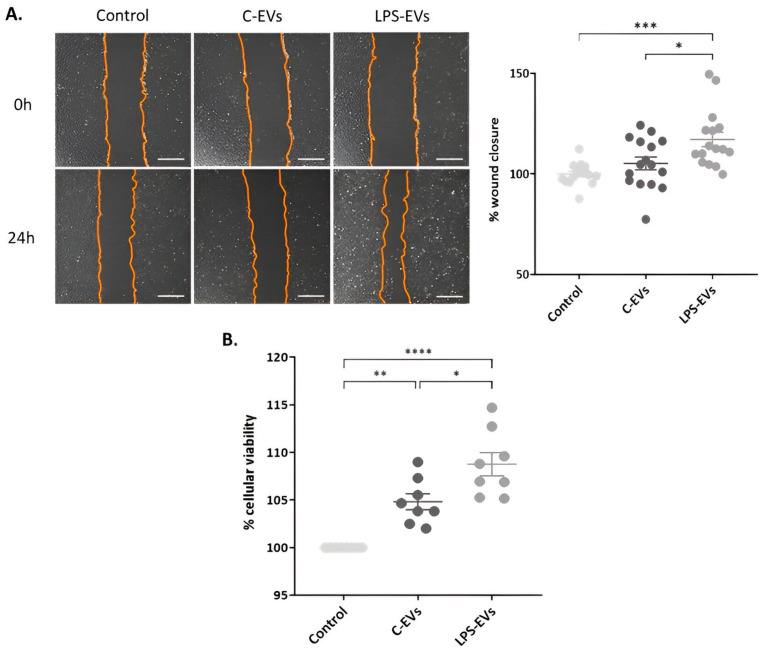
Effect of MSCs-derived EVs on wound healing and cell proliferation in vitro. (**A**) Representative optical images of wound healing in HPAEpiC monolayer. Magnification: 20×. Scale bar: 50 µm. Percentage of wound closure in HPAEpiC 24 h after being treated with C-EVs and LPS-EVs. (**B**) Percentage of cell viability of HPAEpiC 24 h after being treated with C-EVs and LPS-EVs, considering that non-treated cells (control) had 100% cellular viability. Data are presented as mean ± SEM of eight (**A**) and four (**B**) independent experiments with two replicates of each condition; * *p* < 0.05; ** *p* < 0.01; *** *p* < 0.001; **** *p* < 0.0001. Abbreviations: extracellular vesicles, EVs.

Scratch wound assays were performed to determine the effect of MSCs-derived EVs on regeneration. The LPS-EVs treatment showed a significantly accelerated wound healing process compared to the C-EVs treatment in HPAEpiC monolayer. Cells that were treated with LPS-EVs significantly regenerated the wound 117% ± 3.58 in comparison with non-treated cells (*p* = 0.0003), also significantly higher than C-EVs (*p* = 0.013). In contrast, HPAEpiC that received C-EVs only healed the wound 105% ± 3.13 compared to control cells, an increase that was not significant (Figure 3A).

Next, a cell proliferation assay was performed by an MTS assay. Both types of EVs significantly increased cell proliferation, showing 105% ± 0.84 and 109% ± 1.22, respectively, compared to untreated cells (*p* < 0.0001 and *p* = 0.010) (basal growth was set as 100%) (Figure 3B). Again, LPS-EVs produced a significant major effect compared to C-EVs (*p* = 0.002).

### 3.4. LPS-EVs Produce a Major Anti-Inflammatory Effect Compared to C-EVs in P. aeruginosa-Infected Macrophage-like THP-1 Cells In Vitro

**Figure 4:**
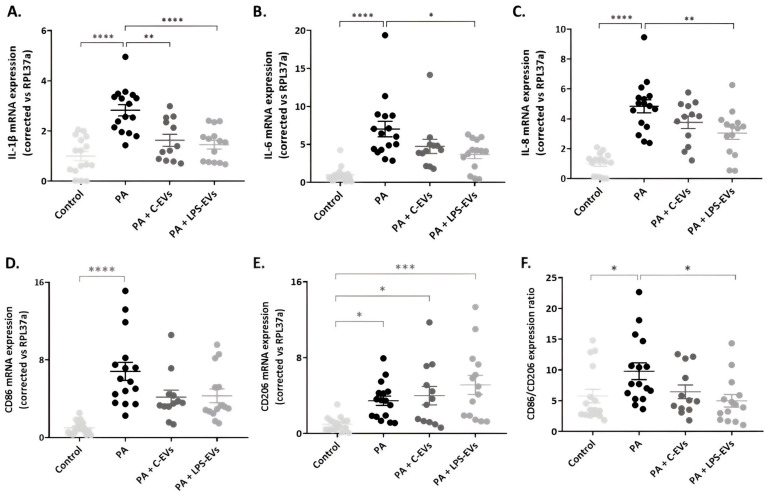
Effect of MSCs-derived EVs on infected THP-1 cells in vitro. Representation of mRNA expression of pro-inflammatory cytokines, chemoattractant mediators, and M1 and M2 macrophage phenotype markers: IL-1β (**A**), IL-6 (**B**), IL-8 (**C**), CD86 (**D**) and CD206 (**E**) in THP-1 cells activated with LPS and infected by *P. aeruginosa* and treated with C-EVs or LPS-EVs. (**F**) Ratio of CD86/CD206 expression (M1/M2 ratio). The relative expression of target genes was normalized to RPL37a expression. Data are presented as mean ± SEM of six independent experiments with two or three replicates of each condition. * *p* < 0.05; ** *p* < 0.01; *** *p* < 0.001; **** *p* < 0.0001. Abbreviations: interleukin, IL; extracellular vesicles, EVs.

The effect of MSCs-derived EVs on inflammation was determined in THP-1 macrophages activated by *P. aeruginosa* infection. Both C-EVs and LPS-EVs significantly decreased the mRNA expression of IL-1β (*p* = 0.001 and *p* < 0.0001) (Figure 4A). Regarding IL-6 and IL-8, EVs also diminished their expressions, but this reduction only reached significant levels in the case of LPS-EVs (*p* = 0.015 and *p* = 0.005) (Figure 4B,C).

The expression of CD86 and CD206, two surface markers characteristic of M1 and M2 macrophage phenotypes, were also evaluated. Macrophage-like THP-1 cells infected with *P. aeruginosa* showed an increased expression of both CD86 and CD206 (*p* < 0.0001 and *p* = 0.033, respectively), with CD86 showing a more substantial increase (7-fold) compared to CD206 (3-fold). However, when THP-1 cells were treated with both types of MSCs-EVs, the expression of CD86 was decreased and the expression of CD206 increased (Figure 4D,E) compared to injured THP-1 macrophages, although the differences were not significant. The ratio of M1/M2 (CD86/CD206) reflects the shift in macrophage phenotype. Injury with *P. aeruginosa* induced a notable increase in this ratio, indicating a shift towards the M1 phenotype, which was attenuated by MSCs-EVs treatment. This reduction only reached significance with LPS-EVs (*p* = 0.024) and, together with a major effect of LPS-EVs on IL-1 β, IL-6 and IL-8, suggested that LPS-EVs have a greater potential in comparison with C-EVs to promote macrophage polarization towards the M2 phenotype.

### 3.5. C-EV and LPS-EV Therapy Ameliorates Lung/Body Weight Ratio In Vivo

**Figure 5:**
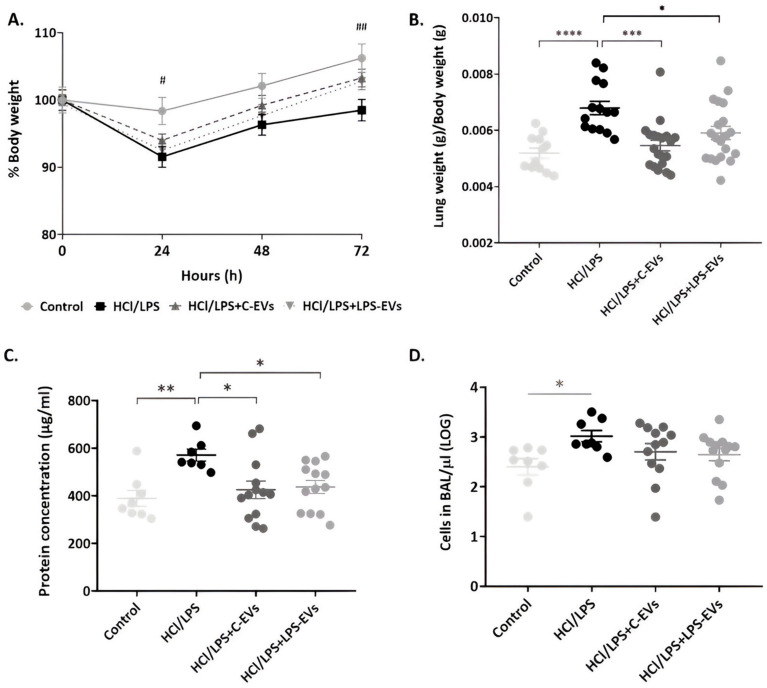
Effect of MSCs-derived EVs on animals’ weight and lung permeability. (**A**) Monitoring of the animals’ body weight every 24 h, considering 100% as the starting body weight for each group (# *p* < 0.05; ## *p* < 0.01 control group vs. HCl + LPS group). (**B**) Ratio of lung weight/body weight measured at the end of the experiment (grams/grams) (*n* = 12–22). (**C**) Total protein concentration (µg/mL) and (**D**) cells’ concentration (cells/µL) in the BAL fluid at the end point (72 h) (*n* = 8–13); Data are presented as mean ± SEM * *p* < 0.05; ** *p* < 0.01; *** *p* < 0.001; **** *p* < 0.0001. Abbreviations: extracellular vesicles, EVs.

The kinetics of body weight variation at 72 h is similar among all groups of animals; animals that received both types of EVs presented attenuated weight loss and better recovery at 72 h compared to injured and untreated animals (Figure 5A). To assess lung damage, the presence of pulmonary injury was measured by the ratio between lung and body weights. As expected, administration of HCl + LPS resulted in a significant increase in the lung/body weight ratio, which was reduced in rats receiving both C-EVs or LPS-EVs (*p* = 0.0003 and *p* = 0.025) (Figure 5B).

### 3.6. LPS-EVs and C-EVs Reduce Lung Permeability, but Only LPS-EVs Significantly Decrease Neutrophil Infiltration In Vivo

Lung permeability was determined by the protein concentration and cell infiltration in the alveolar compartment, which was reflected in the BAL. Animals that were administered with MSCs-EVs significantly reverted the increase in the protein concentration in the BAL of injured animals (572 µg/mL), showing a mean of 425 µg/mL in C-EVs-treated animals and a 438 µg/mL in LPS-EVs-treated animals (*p* = 0.028 and *p* = 0.049) (Figure 5C). In line with these results, cell infiltration of animals treated with MSCs-derived EVs was not as high as in the HCl + LPS group (Figure 5D).

**Figure 6:**
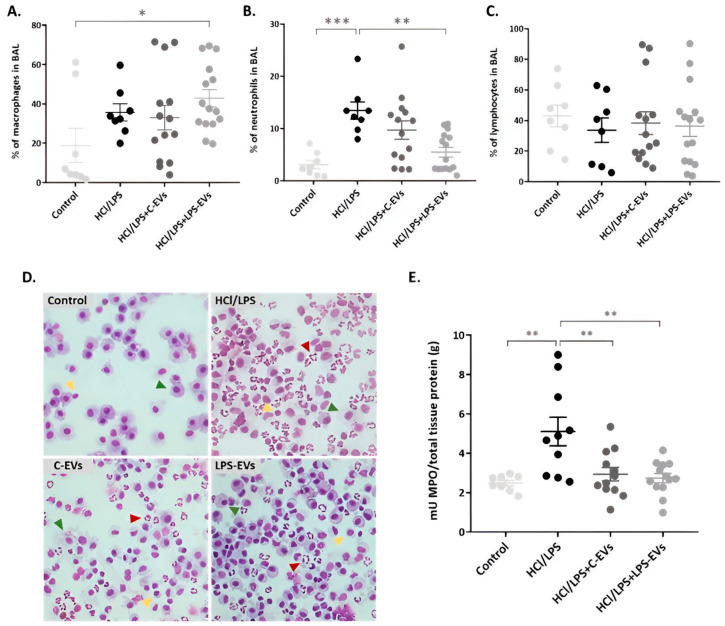
Effect of MSCs-derived EVs on neutrophil infiltration in the intra-alveolar space in vivo at 72 h. Percentage of (**A**) monocytes, (**B**) neutrophils and (**C**) lymphocytes in BAL by flow cytometry. (**D**) Representative images of BAL cells cytospins stained with Diff-Quick. Neutrophils (red arrows), macrophages (green arrows) and lymphocytes (yellow arrows) are indicated in each image. Magnification: 20×. (**E**) MPO activity quantification in lung tissue homogenates (mU/total tissue protein (g)). Data are presented as mean ± SEM (*n* = 8–14); * *p* < 0.05; ** *p* < 0.01; *** *p* < 0.001.

Differential cell counts in BAL revealed a significant increase in the percentages of activated monocytes (CD11b+ HIS48+ cells) in the animals administered with HCl + LPS, both treated and non-treated. Only LPS-EVs exhibited a significant increase, showing a 43.3% ± 4.5 increase compared to the control animals that presented a 18.9% ± 8.7 increase in HIS48+ monocytes (*p* = 0.046) (Figure 6A). Regarding the percentage of neutrophils (CD11b+ and His48++ cells with high granularity) in the BAL, a significant reduction was found in animals that received LPS-EVs in comparison with injured and non-treated animals, showing decreases of 5.7% ± 0.9 and 13.5% ± 1.6, respectively (*p* = 0.004). However, animals that were instilled with C-EVs, exhibiting a 9.7% ± 1.7 decrease in neutrophils in BAL, did not significantly decrease the percentage of neutrophils compared to the injured group (Figure 6B). No substantial changes were observed in the total percentage of lymphocytes (CD45+ and CD11b-) among all the groups (Figure 6C). In addition, the results obtained by flow cytometry were reinforced by BAL cytospins stained with Diff Quick (Figure 6D). Monocytes (or their differentiated macrophage stages), neutrophils and lymphocytes were counted, taking into consideration their cellular and nuclear morphology, as defined in the methods section.

Furthermore, we measured the MPO activity in lung tissue homogenates as an indicator of neutrophil activity. Again, HCl + LPS administration resulted in a significant increase in MPO levels in the lung, reflecting the high activation of neutrophils. When the animals received both types of MSCs-EVs, the MPO levels were significantly reduced compared to the levels of injured animals (5162 mU/g), with a concentration of 2.94 mU/g in the case of C-EVs and 2.77 mU/g in the case of LPS-EVs (*p* = 0.0004 and *p* = 0.002) (Figure 6E).

### 3.7. MSCs-EVs Modulate Lung Inflammation but LPS-EVs Further Decrease AM’s Chemotaxis Activity In Vivo

**Figure 7:**
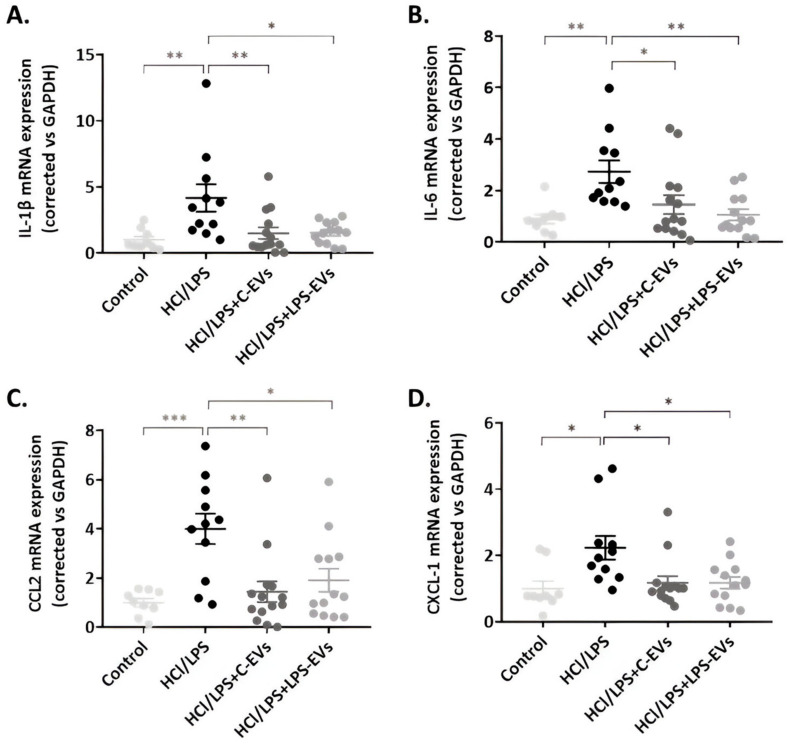
Effect of MSCs-derived EVs on lung tissue inflammation in vivo at 72 h. mRNA expression of pro-inflammatory cytokines, (**A**) IL-1β and (**B**) IL-6, and chemoattractant mediators, (**C**) CCL-2 and (**D**) CXCL-1 (mRNA expression correlated vs. GAPDH). Data are presented as mean ± SEM (*n* = 9–14). * *p* < 0.05; ** *p* < 0.01; *** *p* < 0.001. Abbreviations: interleukin, IL.

The HCl + LPS group at 72 h showed a significant increase in the expression of IL-1β and IL-6, as well as CCL2 and CXCL-1, which are chemotactic mediators of monocytes and neutrophil recruitment, respectively (Figure 7). In contrast, animals treated with both types of EVs exhibited a significantly reduced expression of IL-1β, IL-6, CCL2 and CXCL-1. No differences were found between the animals that were administered with C-EVs and LPS-EVs.

**Figure 8:**
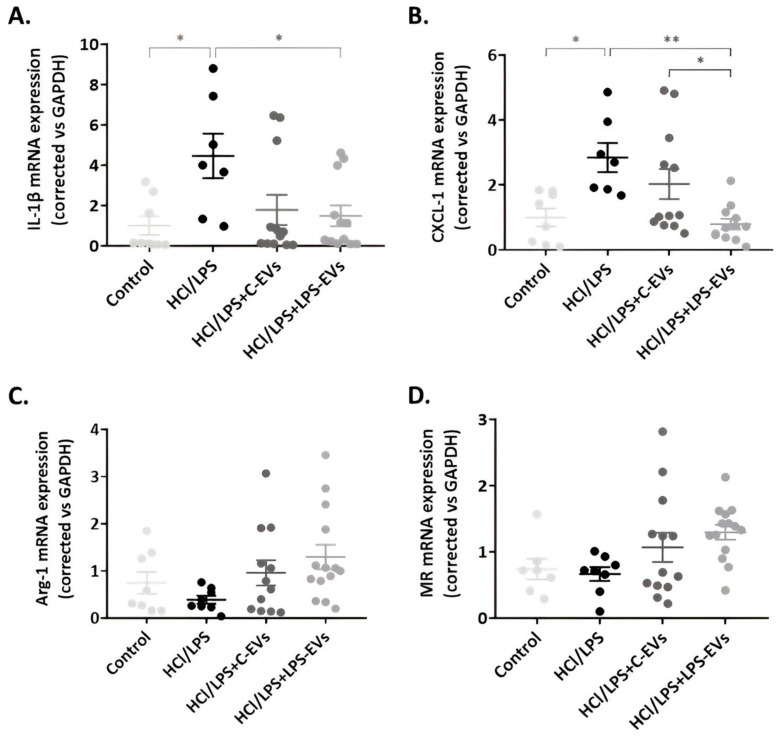
Effect of MSCs-derived EVs on alveolar macrophage inflammation in vivo at 72 h. mRNA expression of pro-inflammatory cytokines, (**A**) IL-1β; chemoattractant mediators, (**B**) CXCL-1; and M2 phenotype markers, (**C**) Arg-1 and (**D**) MR (mRNA expression correlated vs. GAPDH). Data are presented as mean ± SEM (*n* = 8–14). * *p* < 0.05; ** *p* < 0.01. Abbreviations: interleukin, IL; arginase 1, Arg-1; mannose receptor, MR.

In relation to the AM-mediated inflammatory response, the expression of IL-1β and CXCL-1 was augmented in the HCl + LPS group, and their expression was diminished in the animals that were treated with MSCs-EVs, with only significant reductions in animals treated with LPS-EVs (Figure 8A,B). In addition, a significant difference between C-EVs and LPS-EVs was noted in the case of CXCL-1 expression (*p* = 0.004). Regarding alveolar macrophage polarization, both C-EVs and LPS-EVs enhanced the expression of M2 phenotype markers, MR and Arg-1, in AMs, although these increases were not statistically significant (Figure 8C,D).

### 3.8. C-EVs and LPS-EVs Restored Lung Architecture In Vivo

**Figure 9:**
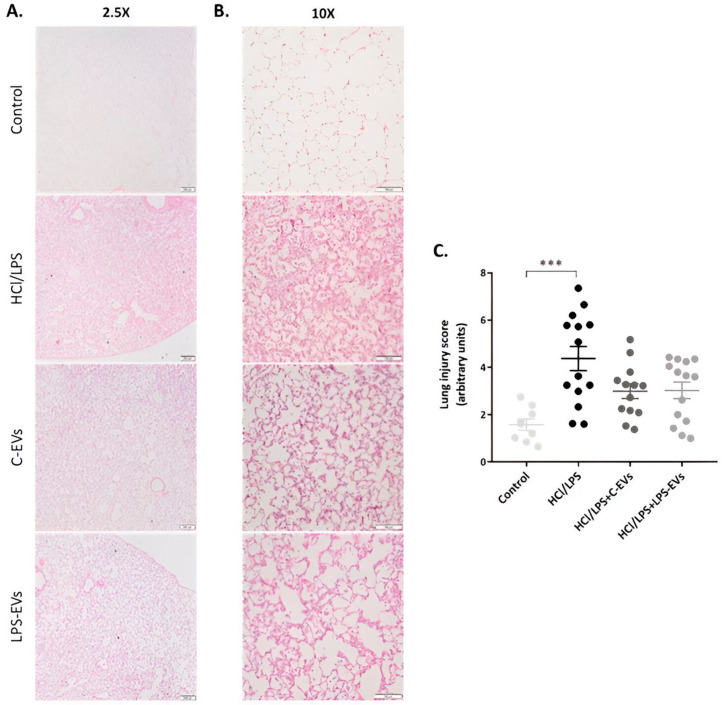
Effect of MSCs-derived EVs on the lung injury in vivo at 72 h. Representative images of H&E histological lung sections of control and injured animals with (**A**) 2.5× and (**B**) 10× magnification. Scale bars: 200 and 100 µm, respectively. (**C**) Quantification of the lung injury score (LIS), evaluating hemorrhage, peribronchial infiltration, interstitial edema, pneumocyte hyperplasia and intra-alveolar infiltration, as described in Supplementary Table S3. Data are presented as mean ± SEM (*n* = 9–14). *** *p* < 0.001.

Several histological lung sections were evaluated, and the representative hallmarks of ALI were quantified using LIS. All sections were screened with a smaller magnification to analyze the extension of the injury and with a higher magnification to focus on the damaged areas (Figure 9B). Analysis of the lung sections at low magnification revealed fewer lung lesions and evidenced large areas of undamaged tissue in animals that were administered with MSCs-EVs therapy (Figure 9A). The images with higher magnification showed reduced edema, hemorrhage, intra-alveolar cell infiltration and pneumocyte hyperplasia in animals that received C-EVs or LPS-EVs compared to injured untreated animals, obtaining a lower overall LIS, although it was not statistically significant (Figure 9C). No significant differences were observed between animals receiving C-EVs and LPS-EVs therapy in the LIS.

## 4. Discussion

In the present study, we report that by subjecting MSCs to environmental stress (LPS priming), the paracrine activity of MSCs is boosted, secreting a higher amount of EVs with an enhanced therapeutic effect. A series of in vitro and in vivo experiments demonstrated that LPS-EVs exhibited a greater regenerative capacity compared to C-EVs, and were able to modulate the acute immune response characteristic of the used ALI models more efficiently than C-EVs. Although the administration of both types of MSCs-EVs decreased epithelial and endothelial permeability and reduced lung injury, LPS-EVs further promoted epithelial regeneration. The infiltration of protein and pro-inflammatory cells in the intra-alveolar space was diminished by both C-EVs and LPS-EVs. However, LPS-EVs restricted neutrophil infiltration to a greater extent and caused a major increase in macrophages with an M2 phenotype.

MSCs that were primed with LPS showed an enhanced paracrine activity as their culture medium contained more EV-like particles and proteins than the media of non-conditioned MSCs. This was previously reported in MSCs primed with cytokines [28,29], hypoxia [30] or 3D cultures [31]. No significant differences were observed either in morphology or in the presence of specific markers between C-EV and LPS-EV populations. The size of both pools of EVs was smaller than 200 nm, which suggests that the isolated particles were exosomes; however, since we cannot specify their subcellular origin, MISEV2023 recommends using the term “small EVs” or better yet, “EVs”, as the measurement of their diameters is related to the specific characterization method used [23]. In this line, we detected a small increase in the mean size of LPS-EVs, which could be in agreement with previous studies where MSCs were also primed with pro-inflammatory factors [32,33]. In addition, despite the multiple washing steps involved in the EVs’ isolation process, the absence of residual LPS molecules attached to EVs cannot be entirely ensured. However, if residual LPS was present, the amount would likely be negligible. This is supported by our findings demonstrating the major immunomodulatory effect of LPS-EVs, both in vitro and in vivo. If the amount of LPS was significant, we would not expect to observe such pronounced effects.

In the pathophysiology of ARDS, multiple pathways of injury, inflammation and coagulation often interplay. Although some potential pharmacotherapeutic agents have demonstrated efficacy in experimental models and clinical trials, the fact that they target only one of the signalling pathways, together with ARDS’s underlying heterogeneity, hinders their efficacy and translation to the clinics [8,34]. In contrast, MSCs-derived EVs exhibit multidirectional functionalities, as they can address different mechanisms underlying the pathophysiology of ARDS.

Our initial in vitro assessment gave great promise to the differential effects of non-primed and primed MSCs’ EVs on tissue healing and innate cell functionality. Our findings highlight the impact of LPS pre-treatment, which resulted in an enhanced wound regenerative capacity and increased proliferation of human alveolar epithelial cells. Although it is well demonstrated that naïve MSC-EVs induce wound regeneration [35], as our results also reflect, LPS-EVs exhibited a superior potential, which is consistent with existing research indicating that the preconditioning of MSCs significantly amplifies their regenerative properties, promoting wound healing and cell proliferation [36,37]. In addition, treating infected macrophages with LPS-EVs decreased their expression of IL-1β, IL-6 and IL-8, and significantly reduced the CD86/CD206 ratio compared to infected and non-treated cells, indicating an attenuation of the acute inflammatory response triggered by the *P. aeruginosa* infection. The immunomodulatory effect of TLR4-stimulated MSCs-EVs has been previously reported in LPS-activated murine AM in vitro [17]. In fact, Forsberg, M.H. et al. demonstrated that EVs derived from TLR-4-stimulated MSCs can modify the genetic expression of the monocytes in vitro, conferring on them a more immunosuppressive and regenerative profile [38]. Furthermore, Ti, D. et al. claimed that the enhanced effect of EVs from LPS-primed MSCs was due to the shuttling of let-7b, a miRNA that has a critical role in regulating macrophage plasticity and, consequently, acting on cutaneous wound healing by targeting the TLR4/NF-κB/STAT3/AKT signalling pathway [36].

M1- and M2-activated macrophages exhibit characteristic transcriptional and secretory profiles, which define their functional roles [39]. In ARDS, the macrophage phenotype fluctuates throughout its phases, allowing them to contribute to both the initial inflammatory response and the resolution of the syndrome [40]. In our in vitro model, infection by *P. aeruginosa* induced a marked increase in CD86 expression, and a slight rise in CD206, suggesting a heterogeneous population of macrophages characterized by high plasticity, as observed in other contexts [39,41]. However, the ratio between both markers (CD86/CD206) clearly indicated a polarization towards the M1 phenotype in infected and non-treated macrophages, which was significantly reduced when cells received LPS-EVs. This, together with a lower expression of pro-inflammatory cytokines, revealed a higher prevalence of M2 macrophages in this group.

Our in vivo ALI model reproduces many of the pathophysiological hallmarks of ARDS, including the disruption of the alveolar–capillary barrier, which increases epithelial and endothelial permeability, leading to an increased protein-rich edema and the presence of pro-inflammatory cells in the alveolar compartment. In the present study, C-EVs and LPS-EVs were able to diminish the total protein concentration in BAL, as well as the total cell infiltration in alveolar and interstitial spaces of the injured animals, indicating that the lung permeability caused by the HCl and LPS administration could be reverted by a partial reestablishment of alveolar barrier integrity. This is in agreement with previous studies also reporting on an attenuated lung permeability when LPS-induced ALI animals received naïve MSCs-EVs [42], MSCs-EVs overexpressing miR-30b-3p [43] or EVs secreted by INF-ɣ-primed MSCs [44]. More specifically, Silva JD, et al. demonstrated that MSCs-EVs restored the alveolar barrier integrity in a mouse model of lung injury by restoring mitochondrial respiration in the lung tissue hampered by the LPS administration [12].

One of the main differences we observed between the effect of C-EVs and LPS-EVs was the percentage of neutrophils in BAL. Only the animals treated with LPS-EVs had a lower neutrophilic infiltration and MPO activity than injured animals, while C-EVs only decreased the MPO activity. This could be explained by a previous demonstration by Munir S, et al., who showed the exposure of MSCs to LPS reprogrammed their transcriptome, playing a significant role in neutrophil activation and their infiltration of injured sites by detecting increased neutrophil extracellular traps (NETs) formation [45]. MPO is a necessary enzyme for the formation of NETs, which are associated with ARDS severity [46], but also are essential for preventing the growth and dissemination of pathogens and for tissue regeneration [47]. Obtained data might suggest that LPS-EVs reduce neutrophil infiltration while enhancing their activity, balancing ALI resolution [48].

The lung inflammatory response is characteristic of ARDS physiopathology. Our model presents increased MCP-1, IL-1β and TNF-α expression in lung tissue [24]. Although the immune cells are the most described in relation to the pulmonary inflammation in ALI, it is well accepted that epithelial cells also play an essential role in the development of the disease. In fact, it has been demonstrated that the intratracheal instillation of LPS can activate airway epithelial cells, prompting them to produce inflammatory factors and initiate the reaction in the first place [49]. In addition, the epithelial alveolar cells, which act as targets and sources of cytokines/chemokines, orchestrate lung inflammation along with activated macrophages [50]. In the present investigation, the effect of MSCs-EVs on mitigating lung tissue inflammation is similar whether MSCs are primed or not, re-establishing lung tissue homeostasis.

However, if we focus specifically on the state of AMs, we found that AMs from animals that were administered with LPS-EVs showed a significantly decreased expression of the chemotactic factor responsible for neutrophil recruitment, CXCL-1, compared to C-EV-treated animals. This reinforces our previously discussed results, proving that LPS-EVs act on the chemotactic state of AMs, being responsible for the significantly reduced percentage of infiltrated neutrophils in BAL. While these results emphasize the boosted chemoattractant activity of LPS-EVs, the M2 polarization of AMs in the BAL did not fully reflect the effects observed in vitro. Although AMs from animals that received MSCs-EVs seemed to increase Arg-1 and MR expression, along with reduced IL-1β expression, these changes were not statistically significant. Nonetheless, these results reinforce previous studies of our group that also outlined the essential role of AMs in the development and resolution of ARDS [51,52].

The outlined data are also reflected in the histological sections, where MSCs-EVs attenuated the loss of epithelial barrier integrity by reducing the presence of edema, hemorrhage, alveolar hyperplasia and cellular infiltration in the interstitial space. However, animals administered C-EVs and LPS-EVs did not show a significant difference in LIS quantification. We hypothesize that this is due to the fact that, although our model is prolonged, allowing for the maintenance of lung injury and inflammation for 72 h, it is actually an experimental model reflecting the acute phase of ARDS, in which the subsequent subacute proliferative phase has not yet started [24]. This suggests that the enhanced effect on tissue regeneration and remodeling of alveolar architecture of LPS-EVs may not yet be noticeable after 72 h. Nevertheless, the fact that LPS-EV-treated animals have a lower infiltration of neutrophils suggests that these animals will have a better resolution of ALI across longer time periods.

Our results highlight a complex interplay between the inherent properties of MSCs-derived EVs and the effects of LPS priming. While it is well established that EVs from naïve MSCs possess therapeutic effects independently, LPS priming appears to enhance certain functions. This indicates a modular response, wherein some therapeutic effects are intrinsic to MSCs-EVs, while others are influenced by LPS treatment. This enhancement could be attributed to the presence of molecular factors induced by priming that specifically improve these functions. Our study highlights the benefits that LPS priming can bring to cell therapy based on the paracrine activity of MSCs in an ALI scenario. However, it has some limitations. We used a two-hit model to reproduce the principal hallmarks of ARDS, but it cannot completely reflect the heterogeneity and complexity of the syndrome [27]. In addition, our ALI model is sterile; it is possible that a more harmful infection model, such as pneumonia or a polymicrobial sepsis model, could take advantage from MSCs-EV therapy differently. In vivo studies were performed in young male rats (7–8 weeks old), which hindered the evaluation of MSCs-EVs’ effectiveness in elderly ARDS patients (≥65 years of age), who are associated with increased morbidity and mortality [53], and also in females, even though it has been demonstrated that ARDS mortality does not differ according to gender [54].

Given the promising results that MSCs-EVs offer, they could be a viable replacement for MSCs therapy, with the additional benefit of being easier to produce and store [13]. Furthermore, characterizing the features and the content of EVs after priming MSCs with LPS will be essential to understand their underlying therapeutic mechanisms. The fact that LPS-primed MSCs-EVs exhibited enhanced therapeutic potential could help us to identify specific bioactive components, responsible for this differential effect. This might be an alternative strategy to overcome some of the translational challenges of the clinical implementation of MSCs and EVs, including optimization of purification and storage methods, quality, reproducibility and the potency of their production [55]. In the future, cell-based but cell-free therapies could be developed, taking advantage of nanotechnology, for instance.

In conclusion, LPS-primed MSCs exhibit a potentiated paracrine activity compared to non-primed MSCs, with an improved immunomodulatory and regenerative effect of their secreted EVs. LPS-EVs have a major influence on alveolar macrophages, triggering their polarization towards an M2 phenotype, which allows them to achieve a balance in infiltration and neutrophil activation, while avoiding the complete disruption of the alveolar barrier, resulting in a better resolution of ALI.

## Data Availability

The datasets generated and/or analyzed during this study are not publicly available as they are included in a pending patent but can be requested from the corresponding author.

## Supplementary Materials

The following supporting information can be downloaded at https://www.mdpi.com/article/10.3390/pharmaceutics16101316/s1: Table S1. Human and rat primers used for real-time polymerase chain reaction. Table S2. List of conjugated antibodies used in the flow cytometry analysis. Table S3. Lung injury score system. Figure S1: Gating strategy examples for flow cytometry analysis for each experimental group. Gated cell populations: Total myeloid cells: CD45+ and CD11b+; Classical monocytes: CD45+ CD11b+ His48+; Neutrophils: CD45+ CD11b+ His48+ with high granularity side scatter (SSC-A).

## Abbreviations

MSCs	Mesenchymal Stem Cells
EVs	Extracellular vesicles
ARDS	Acute Respiratory Distress Syndrome
LPS	Lipopolysaccharide
HCl	Hydrochloric acid
ALI	Acute Lung Injury
TLR4	Toll-like receptor 4
DMEM	Dulbecco’s Modified Eagle Medium
FBS	Fetal bovine serum
PBS	Phosphate-buffered saline
C-EVs	Control-extracellular vesicles
LPS-EVs	LPS-extracellular vesicles
Cryo-TEM	Cryo-transmission electron microscopy
HPAEpiC	Human Pulmonary Alveolar Epithelial cells
THP-1	Human Leukemia Monocytic cell line
RPMI	Roswell Park Memorial Institute medium
PA	*Pseudomonas aeruginosa*
LB	Luria Broth
IL	Interleukin
RT-qPCR	Real-time quantitative reverse transcription polymerase chain reaction
NTA	Nanoparticle tracking analysis
BAL	Bronchoalveolar lavage
ACK	Ammonium chloride potassium
AM	Alveolar macrophages
H&E	Hematoxylin-eosin
LIS	Lung injury score
Arg-1	Arginase-1
MR	Mannose receptor
MPO	Myeloperoxidase
NETs	Neutrophil extracellular traps
HRP	Horseradish peroxidase
SEM	Standard error of the mean
*p*	*p*-value
