# Supplementary material for: Priming Mesenchymal Stem Cells with Lipopolysaccharide Boosts the Immunomodulatory and Regenerative Activity of Secreted Extracellular Vesicles
Source: Pharmaceutics. 2024 Oct 10;16(10):1316. doi: 10.3390/pharmaceutics16101316 (PMC11510928; doi:10.3390/pharmaceutics16101316)
Supplement: Supplementary file 1 [file pharmaceutics-16-01316-s001.zip › pharmaceutics-3219739-supplementary.pdf]

Supplementary material:

Supplementary Table S1. Human and rat primers used for real-time polymerase chain reaction.

GENE	SPICES	FUNCTIONS	FORWARD PRIMER	REVERSE PRIMER
**RPL37A**	Human	Housekeeping	5′ATTGAAATCAGCCAGCACGC3′	5′AGGAACCACAGTGCCAGATCC3′
**GAPDH**	Rat	Housekeeping	5′CTGTGTCTTTCCGCTGTTTTC3′	5′TGTGCTGTGCTTATGGTCTCA3′
**IL-1B**	Human	Pro-inflammatory	5′GGACAAGCTGAGGAAGATGC′	5′TCGTTATCCCATGTGTCGAA3′
**IL-1B**	Rat	Pro-inflammatory	5′AAAAATGCCTCGTGCTGTCT3′	5′TCGTTGCTTGTCTCTCCTTG3′
**IL-6**	Human	Pro-inflammatory	5′TACCCCCAGGAGAAGATTCC3′	5′TTTTCTGCCAGTGCCTCTTT3′
**IL-6**	Rat	Pro-inflammatory	5′CTGCTCTGGTCTTCTGGAGT3′	5′GGTCTTGGTCCTTAGCCACT3′
**IL-8**	Human	Neutrophil recruitment	5′ATTTCTGCAGCTCTGTGTGAA3′	5′TTGTGGATCCTGGCTAGCAGAC3′
**CXCL-1**	Rat	Neutrophil recruitment	5′CCACACTCAAGAATGGTCGC3′	5′GTTGTCAGAAGCCAGCGTTC3′
**CCL2**	Rat	Macrophage recruitment	5′GCTGCTACTCATTCACTGGC3′	5′GGTGCTGAAGTCCTTAGGGT3′
**CD86**	Human	M1 macrophage phenotype marker	5′ATCAAGGACACGGGCTTGTA3′	5′GCATGTTGTCGCCATACTCA3′
**CD206**	Human	M2 macrophage phenotype marker	5′CAGATGCCCGGAGTCAGATC3′	5′TTTATCCACAGCCACGTCCC3′
**ARG-1**	Rat	Anti-inflammatory	5′GGGAAGACACCAGAGGAGGT3′	5′TGATGCCCCAGATGACTTTT3′
**MR**	Rat	Anti-inflammatory	5′CCAGGTGGTTTATGGGATGT3′	5′GGGTTCAGGAGTTGTTGTGG3′

Supplementary Table S2. List of conjugated antibodies used in the flow cytometry analysis.

Antibody	*Fluororchrome*	*Source*	*Reference*	*Dilution*
CD45	PE-Cy7	BioLegend	202214	1:200
CD11b	PE	BioLegend	201807	1:200
His48	FITC	eBioscience	15268119	1:200
CD161	APC	Biolegend	205606	1:100

Supplementary Table S3. Lung injury score system.

Parameter	Score per field
Hemorrhage	0-1
Peribronchial infiltration	0-1
Interstitial edema	0-2
Pneumocyte hyperplasia	0-3
Intra-alveolar infiltration	0-3

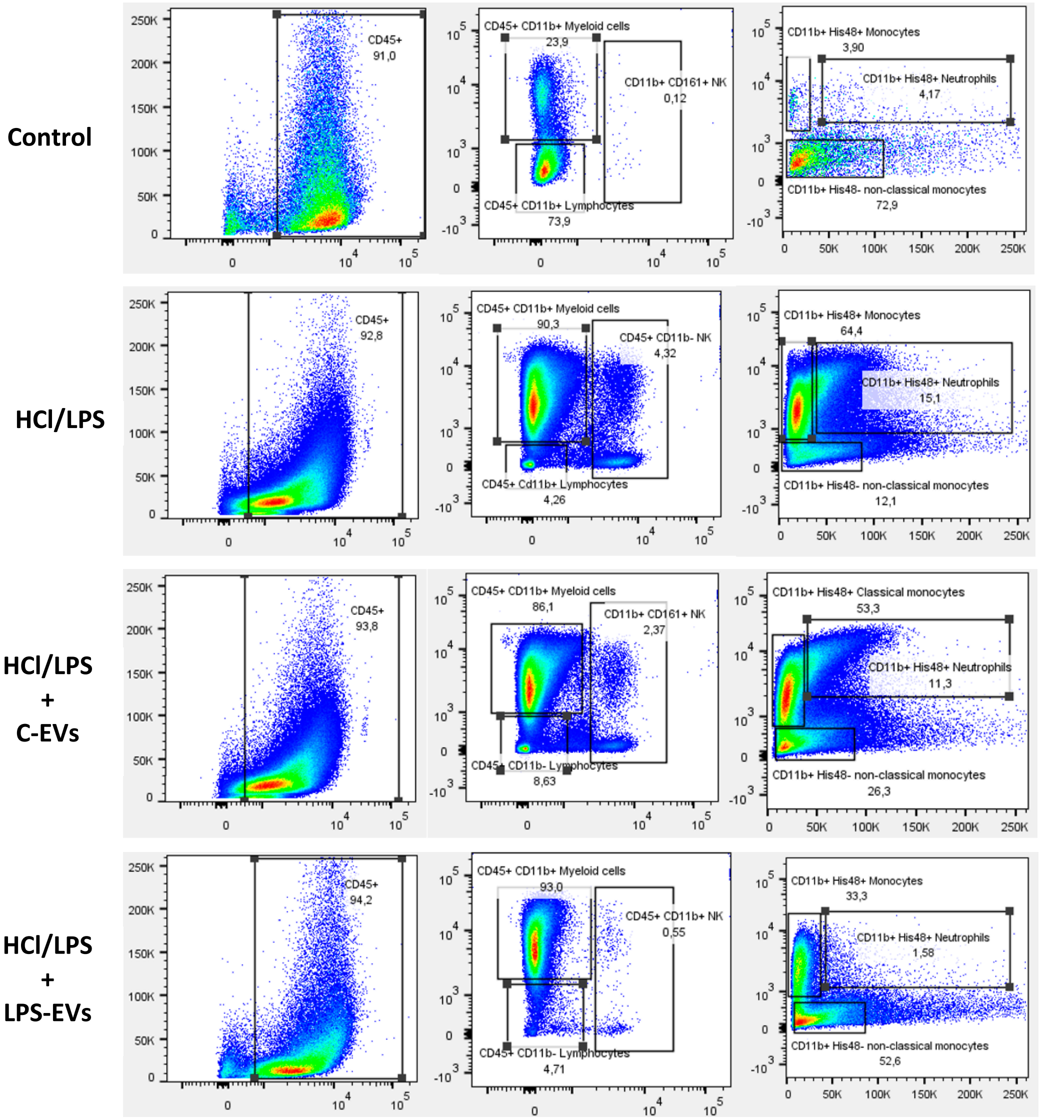

Supplementary Figure S1: Gating strategy examples for flow cytometry analysis for each experimental group. Gated cell populations: Total myeloid cells: CD45+ and CD11b+; Classical monocytes: CD45+ CD11b+ His48+; Neutrophils: CD45+ CD11b+ His48+ with high granularity (side scatter (SSC-A).
